# *catena*-Poly[[tetra­aqua­(μ-4,4′-bipyridine-κ^2^ *N*:*N*′)zinc(II)] fumarate tetra­hydrate]

**DOI:** 10.1107/S1600536808009227

**Published:** 2008-04-16

**Authors:** Yong Tian, Ya-Pan Wu, Dong-Sheng Li, Hui Wang, Ji-Wu Wang

**Affiliations:** a Department of Chemistry, Shaanxi Key Laboratory of Physico-Inorganic Chemistry, Northwest University, Xi’an 710069, People’s Republic of China; b Department of Chemistry and Chemical Engineering, Shaanxi Key Laboratory of Chemical Reaction Engineering, Yan’an University, Yan’an 716000, People’s Republic of China

## Abstract

In the title compound, {[Zn(C_10_H_8_N_2_)(H_2_O)_4_](C_4_H_2_O_4_)·4H_2_O}_*n*_, the Zn^II^ atom is coordinated by two N atoms from two μ-4,4′-bipyridine ligands and four water mol­ecules in a distorted octa­hedral geometry. The coordination unit is extended through the Zn—N bond, leading to a one-dimensional cationic chain. A twofold rotation axis passes through the Zn atom and along the axis of the 4,4′-bipyridine ligand. Each uncoordinated water mol­ecule acts as both hydrogen-bond donor and acceptor. A three-dimensional network is constructed through hydrogen bonds involving water mol­ecules and fumarate dianions.

## Related literature

For related literature, see: Lu *et al.* (2006 ▶); Moulton & Zaworotko (2001 ▶); Nordell *et al.* (2003 ▶); Wagner *et al.* (2002 ▶); Wen *et al.* (2005 ▶); Yaghi *et al.* (1997 ▶); Zaworotko (2001 ▶); Zhou *et al.* (2007 ▶).
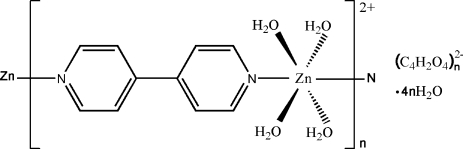


## Experimental

### 

#### Crystal data

[Zn(C_10_H_8_N_2_)(H_2_O)_4_](C_4_H_2_O_4_)·4H_2_O
*M* *_r_* = 479.74
Monoclinic, 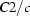

*a* = 17.094 (5) Å
*b* = 11.394 (3) Å
*c* = 13.082 (6) Å
β = 126.652 (2)°
*V* = 2044.3 (12) Å^3^
*Z* = 4
Mo *K*α radiation
μ = 1.26 mm^−1^
*T* = 293 (2) K
0.39 × 0.28 × 0.26 mm

#### Data collection

Bruker SMART APEXII CCD area-detector diffractometer
Absorption correction: multi-scan (*SADABS*; Sheldrick, 1996 ▶) *T*_min_ = 0.626, *T*_max_ = 0.712
7538 measured reflections
1907 independent reflections
1724 reflections with *I* > 2σ(*I*)
*R*_int_ = 0.051

#### Refinement

*R*[*F* ^2^ > 2σ(*F* ^2^)] = 0.040
*wR*(*F* ^2^) = 0.112
*S* = 1.09
1907 reflections
134 parameters
H-atom parameters constrained
Δρ_max_ = 0.98 e Å^−3^
Δρ_min_ = −0.86 e Å^−3^

### 

Data collection: *APEX2* (Bruker, 2007 ▶); cell refinement: *SAINT* (Bruker, 2007 ▶); data reduction: *SAINT*; program(s) used to solve structure: *SHELXS97* (Sheldrick, 2008 ▶); program(s) used to refine structure: *SHELXL97* (Sheldrick, 2008 ▶); molecular graphics: *SHELXTL* (Sheldrick, 2008 ▶); software used to prepare material for publication: *SHELXTL*.

## Supplementary Material

Crystal structure: contains datablocks I, global. DOI: 10.1107/S1600536808009227/hy2122sup1.cif

Structure factors: contains datablocks I. DOI: 10.1107/S1600536808009227/hy2122Isup2.hkl

Additional supplementary materials:  crystallographic information; 3D view; checkCIF report

## Figures and Tables

**Table:** 

Zn1—O2	2.0697 (18)
Zn1—N2^i^	2.133 (3)
Zn1—N1	2.146 (3)
Zn1—O1	2.186 (2)

**Table:** 

O2^ii^—Zn1—O2	179.01 (9)
O2—Zn1—N2^i^	89.50 (4)
O2—Zn1—N1	90.50 (4)
O2—Zn1—O1^ii^	91.97 (7)
O2—Zn1—O1	88.01 (7)
N2^i^—Zn1—O1	89.21 (4)
N1—Zn1—O1	90.79 (4)
O1^ii^—Zn1—O1	178.43 (8)

Symmetry codes: (i) 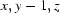; (ii) 
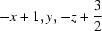
.

**Table 2:** Hydrogen-bond geometry (Å, °)

*D*—H⋯*A*	*D*—H	H⋯*A*	*D*⋯*A*	*D*—H⋯*A*
O1—H1*W*⋯O3^iii^	0.83	1.93	2.757 (2)	173
O1—H2*W*⋯O4^iv^	0.83	2.01	2.835 (3)	172
O2—H3*W*⋯O3	0.83	1.91	2.732 (2)	172
O2—H4*W*⋯O6^v^	0.82	1.83	2.623 (3)	162
O3—H5*W*⋯O5	0.83	1.88	2.707 (3)	173
O3—H6*W*⋯O4^iv^	0.82	2.10	2.911 (3)	172
O4—H7*W*⋯O6^v^	0.83	2.00	2.832 (3)	175
O4—H8*W*⋯O5	0.83	1.99	2.811 (3)	169

Symmetry codes: (iii) 
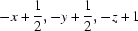
; (iv) 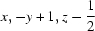; (v) 
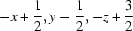
.

**Fig. 1.**
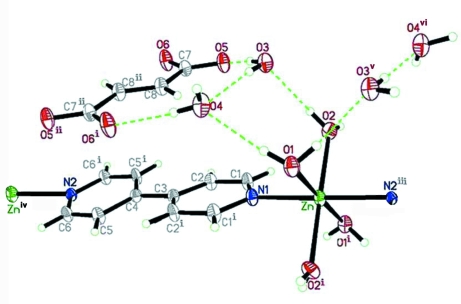
The structure of the title compound. Displacement ellipsoids are drawn at the 30% probability level. [Symmetry codes: (i) 1 - x, y, 3/2 - z; (ii) 1/2 - x, 3/2 - y, 1 - z; (iii) x, -1 + y, z; (iv) x, 1 + y, z; (v) 1/2 - x, 1/2 - y, 1 - z; (vi) 1/2 - x, -1/2 + y, 3/2 - z.]

**Fig. 2.**
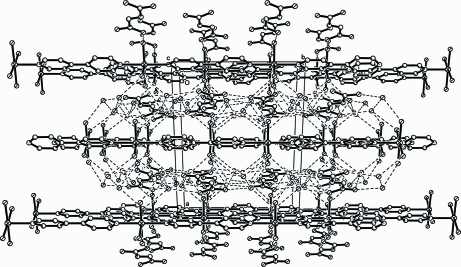
A view of the three-dimensional network in the title compound, viewed down the a axis.

## supplementary crystallographic information

### Comment

Coordination polymer networks represent a result of applying supramolecular concepts to the design of new functional solids and are well exemplified by compounds in which transition metal centers (nodes) are connected by linear bidentate organic ligands (spacer groups) such as 4,4'-bipyridine (Lu *et al.*, 2006; Nordell *et al.*, 2003; Wagner *et al.*, 2002; Wen *et al.*, 2005; Yaghi *et al.*, 1997; Zhou *et al.*, 2007). These supramolecular structures are of interest as they provide opportunity for generating open framework compounds with controllable cavity sizes and they therefore have the potential to exhibit porosity and/or encapsulate guest molecules (Moulton & Zaworotko, 2001; Zaworotko, 2001).

The title compound consists of one [Zn(C_10_H_8_N_2_) (H_2_O)_4_]^2+^ cation, one fumarate dianion and four lattice water molecules (Fig. 1). Each Zn^II^ atom is six-coordinated in an octahedral geometry with four O atoms of four water molecules in the equatorial plane and two N atoms from two µ-4,4'-bipyridine ligands in the axial sites, resulting a one-dimensional cationic chain along the *b*-axis. The two pyridyl rings of 4,4'-bipyridine present a torsion angle of 10.0 (2)°.

Each lattice water molecule acts as both hydrogen-bond donor and acceptor. In the crystal structure, the cationic chains are arranged parallel to the *bc* plane with the fumarate dianions and lattice water molecules located between the sheets composed of the chains. A three-dimensional supramolecular network is formed by hydrogen-bonding interactions involving the water molecules and fumarate dianions (Fig. 2).

### Experimental

A mixture of Zn(NO_3_)_2_.6H_2_O (0.030 g, 0.1 mmol), 4,4'-bipyridine (0.016 g, 0.1 mmol), mercaptosuccinic acid (0.015 g, 0.1 mmol), NaOH (0.008 g, 0.2 mmol) and distilled water (10 ml) was sealed in a 25 ml Teflon-lined stainless autoclave and heated at 433 K for 72 h under autogenous pressure. After slowly cooling to room temperature, yellow block-like crystals of the title compound suitable for X-ray analysis were obtained from the reaction mixture by filtration.

### Refinement

H atoms of the water molecules were located in a difference Fourier map and fixed in the refinements with *U*_iso_(H) = 1.5*U*_eq_(O). The remaining H atoms were positioned geometrically and refined as riding atoms, with C—H = 0.93 Å and *U*_iso_(H) = 1.2*U*_eq_(C)

### Crystal data

**Table:**

[Zn(C_10_H_8_N_2_)(H_2_O)_4_](C_4_H_2_O_4_)·4H_2_O	*F*_000_ = 1000
*M**_r_* = 479.74	*D*_x_ = 1.559 Mg m^−^^3^
Monoclinic, *C*2/*c*	Mo *K*α radiation λ = 0.71073 Å
Hall symbol: -C 2yc	Cell parameters from 3920 reflections
*a* = 17.094 (5) Å	θ = 2.3–28.2º
*b* = 11.394 (3) Å	µ = 1.27 mm^−^^1^
*c* = 13.082 (6) Å	*T* = 293 (2) K
β = 126.652 (2)º	Block, colorless
*V* = 2044.3 (12) Å^3^	0.39 × 0.28 × 0.26 mm
*Z* = 4	

### Data collection

**Table:**

Bruker SMART APEXII CCD area-detector diffractometer	1907 independent reflections
Radiation source: fine-focus sealed tube	1724 reflections with *I* > 2σ(*I*)
Monochromator: graphite	*R*_int_ = 0.051
*T* = 293(2) K	θ_max_ = 25.5º
φ and ω scans	θ_min_ = 2.3º
Absorption correction: multi-scan(SADABS; Sheldrick, 1996)	*h* = −20→20
*T*_min_ = 0.626, *T*_max_ = 0.712	*k* = −13→13
7538 measured reflections	*l* = −15→15

### Refinement

**Table:**

Refinement on *F*^2^	Hydrogen site location: inferred from neighbouring sites
Least-squares matrix: full	H-atom parameters constrained
*R*[*F*^2^ > 2σ(*F*^2^)] = 0.040	*w* = 1/[σ^2^(*F*_o_^2^) + (0.0789*P*)^2^ + 0.2551*P*] where *P* = (*F*_o_^2^ + 2*F*_c_^2^)/3
*wR*(*F*^2^) = 0.112	(Δ/σ)_max_ < 0.001
*S* = 1.09	Δρ_max_ = 0.99 e Å^−^^3^
1907 reflections	Δρ_min_ = −0.86 e Å^−^^3^
134 parameters	Extinction correction: SHELXL97 (Sheldrick, 2008)
Primary atom site location: structure-invariant direct methods	Extinction coefficient: 0
Secondary atom site location: difference Fourier map	

### Fractional atomic coordinates and isotropic or equivalent isotropic displacement parameters (Å^2^)

**Table:**

	*x*	*y*	*z*	*U*_iso_*/*U*_eq_	
Zn1	0.5000	0.27603 (3)	0.7500	0.02705 (18)	
O1	0.41826 (14)	0.27340 (13)	0.54278 (17)	0.0358 (4)	
H1W	0.3814	0.2172	0.5259	0.054*	
H2W	0.3864	0.3328	0.5041	0.054*	
O2	0.36784 (13)	0.27446 (13)	0.72328 (17)	0.0358 (4)	
H3W	0.3232	0.3120	0.6610	0.054*	
H4W	0.3621	0.2706	0.7812	0.054*	
O3	0.21520 (12)	0.40439 (15)	0.53213 (15)	0.0396 (4)	
H5W	0.2070	0.4658	0.5581	0.059*	
H6W	0.2331	0.4167	0.4872	0.059*	
O4	0.29343 (13)	0.53785 (16)	0.89255 (16)	0.0454 (5)	
H7W	0.3041	0.4667	0.8933	0.068*	
H8W	0.2635	0.5646	0.8191	0.068*	
O5	0.19945 (14)	0.59997 (17)	0.63613 (17)	0.0437 (4)	
O6	0.17990 (18)	0.79445 (17)	0.6206 (2)	0.0499 (5)	
N1	0.5000	0.4644 (2)	0.7500	0.0305 (6)	
N2	0.5000	1.0888 (2)	0.7500	0.0281 (6)	
C1	0.47776 (19)	0.5262 (2)	0.8170 (2)	0.0375 (6)	
H1A	0.4613	0.4854	0.8633	0.045*	
C2	0.47816 (19)	0.6469 (2)	0.8206 (2)	0.0352 (5)	
H2A	0.4638	0.6856	0.8702	0.042*	
C3	0.5000	0.7114 (3)	0.7500	0.0288 (7)	
C4	0.5000	0.8413 (3)	0.7500	0.0286 (7)	
C5	0.5361 (2)	0.9065 (2)	0.6958 (3)	0.0378 (6)	
H5	0.5612	0.8681	0.6584	0.045*	
C6	0.53453 (18)	1.0269 (2)	0.6974 (2)	0.0358 (5)	
H6	0.5587	1.0677	0.6601	0.043*	
C7	0.20633 (18)	0.6996 (2)	0.6001 (2)	0.0330 (5)	
C8	0.24700 (18)	0.7029 (2)	0.5263 (2)	0.0355 (5)	
H8	0.2699	0.6328	0.5167	0.043*	

### Atomic displacement parameters (Å^2^)

**Table:**

	*U*^11^	*U*^22^	*U*^33^	*U*^12^	*U*^13^	*U*^23^
Zn1	0.0401 (3)	0.0197 (3)	0.0348 (3)	0.000	0.0296 (2)	0.000
O1	0.0500 (10)	0.0311 (10)	0.0364 (9)	−0.0015 (6)	0.0313 (8)	0.0004 (6)
O2	0.0455 (10)	0.0390 (11)	0.0419 (10)	0.0077 (7)	0.0362 (8)	0.0091 (7)
O3	0.0540 (10)	0.0334 (9)	0.0408 (9)	−0.0009 (7)	0.0334 (8)	−0.0006 (7)
O4	0.0691 (12)	0.0338 (10)	0.0394 (10)	0.0009 (8)	0.0357 (9)	0.0017 (7)
O5	0.0722 (12)	0.0362 (10)	0.0497 (10)	−0.0015 (9)	0.0509 (10)	0.0025 (8)
O6	0.0879 (15)	0.0365 (10)	0.0668 (13)	−0.0006 (9)	0.0685 (13)	0.0000 (9)
N1	0.0468 (15)	0.0214 (13)	0.0374 (14)	0.000	0.0327 (13)	0.000
N2	0.0357 (13)	0.0213 (13)	0.0346 (14)	0.000	0.0250 (12)	0.000
C1	0.0596 (15)	0.0256 (12)	0.0471 (14)	−0.0003 (11)	0.0424 (13)	0.0025 (11)
C2	0.0574 (14)	0.0249 (11)	0.0435 (13)	0.0034 (10)	0.0410 (12)	−0.0010 (10)
C3	0.0341 (16)	0.0228 (17)	0.0319 (16)	0.000	0.0211 (14)	0.000
C4	0.0354 (15)	0.0247 (16)	0.0308 (15)	0.000	0.0224 (13)	0.000
C5	0.0595 (15)	0.0249 (12)	0.0547 (15)	0.0010 (11)	0.0480 (13)	−0.0029 (11)
C6	0.0528 (13)	0.0271 (12)	0.0489 (14)	0.0000 (10)	0.0418 (12)	0.0019 (10)
C7	0.0462 (13)	0.0339 (12)	0.0312 (11)	−0.0023 (10)	0.0297 (11)	−0.0014 (10)
C8	0.0528 (14)	0.0326 (11)	0.0401 (13)	0.0016 (10)	0.0381 (12)	−0.0007 (10)

### Geometric parameters (Å, °)

**Table:**

Zn1—O2^i^	2.0697 (18)	N2—C6	1.344 (3)
Zn1—O2	2.0697 (18)	N2—C6^i^	1.344 (3)
Zn1—N2^ii^	2.133 (3)	N2—Zn1^iii^	2.133 (3)
Zn1—N1	2.146 (3)	C1—C2	1.376 (3)
Zn1—O1^i^	2.186 (2)	C1—H1A	0.9300
Zn1—O1	2.186 (2)	C2—C3	1.394 (3)
O1—H1W	0.8300	C2—H2A	0.9300
O1—H2W	0.8259	C3—C2^i^	1.394 (3)
O2—H3W	0.8283	C3—C4	1.480 (5)
O2—H4W	0.8210	C4—C5	1.400 (3)
O3—H5W	0.8263	C4—C5^i^	1.400 (3)
O3—H6W	0.8200	C5—C6	1.372 (4)
O4—H7W	0.8299	C5—H5	0.9300
O4—H8W	0.8315	C6—H6	0.9300
O5—C7	1.261 (3)	C7—C8	1.490 (3)
O6—C7	1.261 (3)	C8—C8^iv^	1.312 (5)
N1—C1^i^	1.345 (3)	C8—H8	0.9300
N1—C1	1.345 (3)		
			
O2^i^—Zn1—O2	179.01 (9)	C6—N2—Zn1^iii^	121.70 (14)
O2^i^—Zn1—N2^ii^	89.50 (4)	C6^i^—N2—Zn1^iii^	121.70 (14)
O2—Zn1—N2^ii^	89.50 (4)	N1—C1—C2	123.2 (2)
O2^i^—Zn1—N1	90.50 (4)	N1—C1—H1A	118.4
O2—Zn1—N1	90.50 (4)	C2—C1—H1A	118.4
N2^ii^—Zn1—N1	179.999 (1)	C1—C2—C3	120.1 (2)
O2^i^—Zn1—O1^i^	88.01 (7)	C1—C2—H2A	119.9
O2—Zn1—O1^i^	91.97 (7)	C3—C2—H2A	119.9
N2^ii^—Zn1—O1^i^	89.21 (4)	C2^i^—C3—C2	116.4 (3)
N1—Zn1—O1^i^	90.79 (4)	C2^i^—C3—C4	121.79 (14)
O2^i^—Zn1—O1	91.98 (7)	C2—C3—C4	121.79 (14)
O2—Zn1—O1	88.01 (7)	C5—C4—C5^i^	115.9 (3)
N2^ii^—Zn1—O1	89.21 (4)	C5—C4—C3	122.05 (15)
N1—Zn1—O1	90.79 (4)	C5^i^—C4—C3	122.05 (15)
O1^i^—Zn1—O1	178.43 (8)	C6—C5—C4	120.3 (2)
Zn1—O1—H1W	99.4	C6—C5—H5	119.9
Zn1—O1—H2W	116.6	C4—C5—H5	119.9
H1W—O1—H2W	110.6	N2—C6—C5	123.5 (2)
Zn1—O2—H3W	115.7	N2—C6—H6	118.3
Zn1—O2—H4W	124.3	C5—C6—H6	118.3
H3W—O2—H4W	112.8	O6—C7—O5	124.5 (2)
H5W—O3—H6W	112.2	O6—C7—C8	118.8 (2)
H7W—O4—H8W	110.5	O5—C7—C8	116.7 (2)
C1^i^—N1—C1	116.8 (3)	C8^iv^—C8—C7	124.9 (3)
C1^i^—N1—Zn1	121.60 (14)	C8^iv^—C8—H8	117.5
C1—N1—Zn1	121.60 (14)	C7—C8—H8	117.5
C6—N2—C6^i^	116.6 (3)		
			
O2^i^—Zn1—N1—C1^i^	45.69 (14)	C1—C2—C3—C4	179.23 (18)
O2—Zn1—N1—C1^i^	−134.31 (14)	C2^i^—C3—C4—C5	−10.02 (17)
O1^i^—Zn1—N1—C1^i^	133.71 (14)	C2—C3—C4—C5	169.98 (17)
O1—Zn1—N1—C1^i^	−46.29 (14)	C2^i^—C3—C4—C5^i^	169.97 (17)
O2^i^—Zn1—N1—C1	−134.31 (14)	C2—C3—C4—C5^i^	−10.02 (17)
O2—Zn1—N1—C1	45.69 (14)	C5^i^—C4—C5—C6	−0.19 (18)
O1^i^—Zn1—N1—C1	−46.29 (15)	C3—C4—C5—C6	179.81 (18)
O1—Zn1—N1—C1	133.70 (15)	C6^i^—N2—C6—C5	−0.21 (19)
C1^i^—N1—C1—C2	−0.83 (19)	Zn1^iii^—N2—C6—C5	179.80 (19)
Zn1—N1—C1—C2	179.17 (19)	C4—C5—C6—N2	0.4 (4)
N1—C1—C2—C3	1.6 (4)	O6—C7—C8—C8^iv^	−3.9 (5)
C1—C2—C3—C2^i^	−0.77 (18)	O5—C7—C8—C8^iv^	174.9 (3)

Symmetry codes: (i) −*x*+1, *y*, −*z*+3/2; (ii) *x*, *y*−1, *z*; (iii) *x*, *y*+1, *z*; (iv) −*x*+1/2, −*y*+3/2, −*z*+1.

### Hydrogen-bond geometry (Å, °)

**Table:**

*D*—H···*A*	*D*—H	H···*A*	*D*···*A*	*D*—H···*A*
O1—H1W···O3^v^	0.83	1.93	2.757 (2)	173
O1—H2W···O4^vi^	0.83	2.01	2.835 (3)	172
O2—H3W···O3	0.83	1.91	2.732 (2)	172
O2—H4W···O6^vii^	0.82	1.83	2.623 (3)	162
O3—H5W···O5	0.83	1.88	2.707 (3)	173
O3—H6W···O4^vi^	0.82	2.10	2.911 (3)	172
O4—H7W···O6^vii^	0.83	2.00	2.832 (3)	175
O4—H8W···O5	0.83	1.99	2.811 (3)	169

Symmetry codes: (v) −*x*+1/2, −*y*+1/2, −*z*+1; (vi) *x*, −*y*+1, *z*−1/2; (vii) −*x*+1/2, *y*−1/2, −*z*+3/2.
